# Multivariate Meta-Analysis of Preference-Based Quality of Life Values in Coronary Heart Disease

**DOI:** 10.1371/journal.pone.0152030

**Published:** 2016-03-24

**Authors:** Jelena Stevanović, Petros Pechlivanoglou, Marthe A. Kampinga, Paul F. M. Krabbe, Maarten J. Postma

**Affiliations:** 1 University of Groningen, Department of Pharmacy, Unit of Pharmacoepidemiology and Pharmacoeconomics (PE2), Groningen, The Netherlands; 2 Toronto Health Economics and Technology Assessment (THETA), Toronto, Canada; 3 University of Toronto, Faculty of Medicine, Institute of Health Policy, Management and Evaluation, Toronto, Canada; 4 University of Groningen, University Medical Center Groningen, Department of Cardiology, Thorax Center, Groningen, The Netherlands; 5 University of Groningen, University Medical Centre Groningen, Department of Epidemiology, Groningen, The Netherlands; McGill University Health Center / Royal Victoria, CANADA

## Abstract

**Background:**

There are numerous health-related quality of life (HRQol) measurements used in coronary heart disease (CHD) in the literature. However, only values assessed with preference-based instruments can be directly applied in a cost-utility analysis (CUA).

**Objective:**

To summarize and synthesize instrument-specific preference-based values in CHD and the underlying disease-subgroups, stable angina and post-acute coronary syndrome (post-ACS), for developed countries, while accounting for study-level characteristics, and within- and between-study correlation.

**Methods:**

A systematic review was conducted to identify studies reporting preference-based values in CHD. A multivariate meta-analysis was applied to synthesize the HRQoL values. Meta-regression analyses examined the effect of study level covariates age, publication year, prevalence of diabetes and gender.

**Results:**

A total of 40 studies providing preference-based values were detected. Synthesized estimates of HRQoL in post-ACS ranged from 0.64 (Quality of Well-Being) to 0.92 (EuroQol European”tariff”), while in stable angina they ranged from 0.64 (Short form 6D) to 0.89 (Standard Gamble). Similar findings were observed in estimates applying to general CHD. No significant improvement in model fit was found after adjusting for study-level covariates. Large between-study heterogeneity was observed in all the models investigated.

**Conclusions:**

The main finding of our study is the presence of large heterogeneity both within and between instrument-specific HRQoL values. Current economic models in CHD ignore this between-study heterogeneity. Multivariate meta-analysis can quantify this heterogeneity and offers the means for uncertainty around HRQoL values to be translated to uncertainty in CUAs.

## Introduction

A large number of health-related quality of life (HRQoL) measures for patients with coronary heart disease (CHD) is available in the literature[1–4]. Those measures either describe the HRQoL of patients suffering from CHD overall or distinguish across patients suffering from one of the underlying forms of CHD, specifically stable angina or post-acute coronary syndrome (post-ACS). This interest in estimating the level of HRQoL in CHD is mainly due to its increasing economic and clinical burden [5], the number of CHD prevention and treatment strategies available, and the necessity to assess the impact of a treatment on HRQoL for use as input parameter in cost-utility analyses (CUAs) [6,7].

From the abundance of HRQoL measurements in CHD, only the preference-based HRQoL values can be directly applied in CUA [6,8]. These values express the individual’s preference for living with CHD compared to other health states on an interval scale where the value of zero is assigned to death, and the value of one to full health [7]. Preference-based values can be generated with direct elicitation techniques, such as the time trade-off (TTO), the standard gamble (SG) and the rating scale (RS). Another approach is based on multi-attribute questionnaires, such as the EuroQol 5D (EQ-5D), Short form 6D (SF-6D), health utility index (HUI) and the quality of well-being (QWB) scale [7].

There is significant variation in the published literature with respect to the preference-based HRQoL value of patients with CHD [1,9–11]. Methodological differences between direct preference-based techniques such as the exact specifications of the questions on individuals’ preferences, have been shown to have considerable impact on HRQoL values [7]. Some of the differences in HRQoL values measured with preference-based multi-attribute instruments reflect the variation across instruments on the sensitivity of different health attributes across different severity levels as well as the use of different direct valuation techniques [7]. Finally, HRQoL values can largely vary due to differences in study-level covariates such as patients’ characteristics (e.g. underlying CHD forms, age, and comorbidities), types of treatment applied or time points of measuring HRQoL relative to the disease onset or treatment initiation.

All the aforementioned differences in the underlying methodology indicate that the choice of the HRQoL value to be applied in CUA needs to consider the impact of both instrument-specific properties and study-level covariates. Notably, when selecting a HRQoL value from the published literature, an evidence synthesis can provide better estimates around the mean and variance of HRQoL to inform CUA than a single study value. The application of meta-analysis on HRQoL values is straightforward when all values are measured with the same instrument. However, a number of studies provide multiple and correlated HRQoL values measured in the same population but using different instruments. Conducting separate univariate meta-analyses for each HRQoL is inappropriate as ignoring the within-study correlation might lead to biased mean and standard error (SE) estimates [12,13]. Instead, multivariate meta-regression analysis is recommended [12,13]. Therefore, in this study we aim to systematically summarize and synthesize the published preference-based HRQoL values in CHD and its underlying disease-subgroups (i.e. stable angina and post-ACS) for developed countries. To account for the underlying differences in the instruments used to measure the HRQoL, and the correlation between instrument-specific values both within and between studies, the synthesis of values is conducted on an instrument-specific level. Additionally, the impact of study-level covariates on HRQoL is explored in regression analysis.

## Methods

### Data collection

This study was conducted following the PRISMA guidelines and a checklist for their application in provided in S1 Appendix. Relevant studies reporting HRQoL in CHD were independently screened and systematically reviewed by two team members [JS and PP]. Studies were searched using MEDLINE and EMBASE. The search terms used were: (“coronary disease” or “coronary heart disease” or “myocardial infarction” or “angina” or “acute coronary syndrome”) and (“utility” or “quality of life” or “outcome assessment”) and ("Health Utilities Index" or "quality of well-being" or "rating scale" or "standard gamble" or "time trade-off" or "15D" or "SF-6D" or "EQ-5D" or "HALex"). See S2 Appendix for details of search strategy for MEDLINE and EMBASE. The search was limited to studies applying to developed countries published between 1990 and November 2014. The last search was conducted on the 15^th^ of December 2014. By including only studies from developed countries, we aimed to limit the variation on HRQoL associated with the relation between socio-economic status and HRQoL [14]. Additionally, the references of the identified articles and other systematic reviews were searched for relevant studies not included in the above-mentioned databases (snowballing). Studies were considered eligible for this analysis if they: 1) applied a preference-based instrument of measuring HRQoL (TTO, SG, RS, EQ-5D, SF-6D, SF-15D, HUI, QWB and HALex) 2) reported mean preference-based HRQoL values measured three months or more after the initiation of CHD-treatment or after the onset of CHD, 3) reported standard deviations (SDs) and sample sizes or confidence intervals (CIs)/SEs of those measurements. Duplicate studies were excluded as well as editorials, letters, clinical conference abstracts, reviews and studies that reported median but not mean HRQoL values. Disagreements were resolved through discussion.

Data from the eligible studies were extracted in duplicate by two team members [JS and PP]. Any uncertainties were resolved by discussion. Data were extracted regarding study origin (authors, publication year, country), study design, participants (age, study sample, percentage of men, percentage of diabetics, underlying form of CHD) as well as the HRQoL (mean value, SD or CI/SE of the mean), type of instrument for measuring HRQoL and correlation coefficients between instrument-specific values. Moreover, for the HRQoL measured with the EQ-5D, we recorded a ‘tariff’ applied to the EQ-5D questionnaire data to generate preferences. Tariffs present valuation sets for EQ-5D health states derived using the TTO technique in nationality-specific population samples [7].

### Assumptions and data adjustments

We distinguished between two underlying CHD subgroups: stable angina and post-ACS. The stable angina and the post-ACS groups comprised of stable angina patients and patients with unstable angina or myocardial infarction, respectively, in whom HRQoL was measured at least three months after diagnosis or treatment initiation. Our analysis was limited to HRQoL values measured three months after onset of CHD or treatment initiation hypothesizing that the impact of the acute disease onset or a treatment effect on HRQoL will be stabilized by then. Additionally, all the values identified, including the ones subgrouped to stable angina, post-ACS and the ones for which information on patients’ characteristics were insufficient to provide their allocation to either of the two underlying CHD subgroups, were analysed together as a general CHD group.

Furthermore, no distinction was made between the HRQoL values measured in patient subgroups other than the ones reflecting underlying CHD subgroups (e.g. different treatment or socio-economic subgroups). In cases where studies provided HRQoL values in a subgroup classification that was not of interest, the HRQoL values across subgroups were synthesized to provide a weighted mean and variance estimate for a specific CHD form per study.

### Statistical model

A multivariate meta-analysis was used to estimate synthesized, instrument-specific HRQoL estimates in post-ACS, stable angina and general CHD [12]. This approach accounts for the correlation (both within and between studies) between HRQoL values assessed with different instruments and was extended to a multivariate meta-regression analysis to account for the impact of study-level covariates where appropriate. The general structure of the model applied is presented below in matrix form.

$$\;\gamma_{i}=\;X_{i}\beta_{i}+\delta_{i}+\;\varepsilon_{i}\;i=1,\ldots ,n$$ (1)

Here ***y***_*i*_ stands for a vector of size *p* whose elements comprise the instrument-specific HRQoL values for study *i*, ***X***_*i*_ is a matrix of *p* instruments and *k* covariates, ***β***_*i*_ is the vector of regression coefficients of size *k*, ***δ***_*i*_ is a vector of random-effects terms of size *p* and ***ε***_*i*_ is a vector of random sampling errors of size *p*. We assumed that ***δ***_*i*_~MVN(0,**Δ**), where

$$\Delta =\;\left[\begin{array}{ccc} \tau_{1}^{2} & \cdots & \rho_{\tau \left(jj^{\prime }\right)}\tau_{1}\tau_{p}\\ \vdots & \ddots & \vdots \\ \rho_{\tau \left(jj^{\prime }\right)}\tau_{1}\tau_{p} & \cdots & \tau_{p}^{2} \end{array}\right].$$ (2)

Here Δ represents the between-study variance–covariance matrix and its elements are ${\;\tau }_{p}^{2}$, the between-study instrument-specific variance, and *ρ*_*τ(jj′)*_, the between-study correlation coefficient assessed when measuring the HRQoL values with *j* and *j*′ instruments. Additionally, it was assumed that ***ε***_*i*_~MVN(0,***S***_*i*_), where

$$S_{i}=\;\left[\begin{array}{ccc} \sigma_{i1}^{2} & \cdots & \rho_{jj^{\prime }}\sigma_{i1}\sigma_{ip}\\ \vdots & \ddots & \vdots \\ \rho_{jj^{\prime }}\sigma_{i1}\sigma_{ip} & \cdots & \sigma_{ip}^{2} \end{array}\right]$$ (3)

The matrix ***S***_*i*_ is the within-study variance–covariance matrix with elements $\sigma_{ip}^{2}$, the within-study instrument-specific variance and *ρ*_*jj′*_, the within-study correlation coefficient.

A common problem in multivariate meta-regression is the presence of missing data for variables of interest in eligible studies. In our analysis, missing data were anticipated for some of the ***y***_*i*_ elements and their corresponding variances, as well as for some within-study correlation coefficients. Missing values in ***y***_*i*_ may occur when HRQoL in a particular study was not estimated with all the instruments included in the meta-regression. We resolved this by setting the missing values in ***y***_*i*_ as equal to zero and their corresponding variances equal to an arbitrary large number (1,000) (11). In this way the contribution of these values to the summarized HRQoL estimate was insignificant. The problem related to missing values of *ρ*_*jj′*_ was resolved by retrieving correlation estimates from a more general population without severe comorbidities. Elements of *ρ*_*jj′*_ that still remained missing were assumed to be equal to zero [13]. In order to observe the impact of ignoring the presence of correlation and to account for possibly different values of correlation coefficients, we undertook a sensitivity analysis by assuming correlation coefficients to take values of 0 and 0.5 [15].

In the regression analysis, the covariates incorporated in ***X***_*i*_ were examined for their statistical significance and their impact on reducing some of the between-study heterogeneity on those HRQoL values. Age, publication year, prevalence of diabetes and gender were the covariates examined. Following reasons were employed when choosing covariates: age and gender were previously shown to impact HRQoL [16]; diabetes is a commonly present comorbidity in patients with CHD, and is indicated to be associated with a reduced level of HRQoL (Coffey et al, 2002)[17]; publication year was explored to assess a possible improvement in HRQoL through time. There are no guidelines to suggest the minimal number of studies per outcome of interest (i.e. an instrument-specific HRQoL value) required for the regression analysis to be plausible in the multivariate setting. For this reason, we adopted the guidelines for the univariate setting that suggest a data set sample size of approximately 10 measurements may be sufficient for conducting a regression analysis with one covariate at a time [15]. The regression analysis was limited to those outcomes where at least 10 measurements were available.

In order to indicate the extent of heterogeneity in the true population level of HRQoL that is unexplained by study-level covariates and random sampling error in the multivariate setting, we calculated the $I_{R}^{2}$ and $I_{H}^{2}\;$ statistics, recently suggested by Jackson et al [18]. We used $I_{H}^{2}$ to measure the impact heterogeneity for all HRQoL estimates jointly and the $I_{R}^{2}$ statistic to measure the level of heterogeneity both jointly and separately for instrument-specific HRQoL estimates [18]. For the estimation of the ${\;I}_{R}^{2}$, the *R*^2^ statistic was used as a basis. In the multivariate setting, the *R*^2^ statistic can be interpreted as the ratio of the volumes of CIs for summarized estimates under the random effect model and the volumes of CIs for summarized estimates under the fixed effects models [18]. Under such a notation of the *R*^2^ statistic, Jackson et al. suggested that the $I_{R}^{2}$ can be estimated as $I_{R}^{2}\;=\;\left(R^{2}-\left.1\right)\right./R^{2}$ [18]. Furthermore, the $I_{H}^{2}$ statistic, was estimated as $\;I_{H}^{2}\;=\;\left(H^{2}-\left.1\right)\right./H^{2}$, where the *H*^2^ statistic represents the ratio of a generalized version of Cohran’s Q statistic and its associated degrees of freedom [18].

The multivariate meta-regression analysis was performed using the package mvmeta [19] in the statistical software R (version 2.15.3) [20].

## Results

### Study characteristics

**Fig 1:**
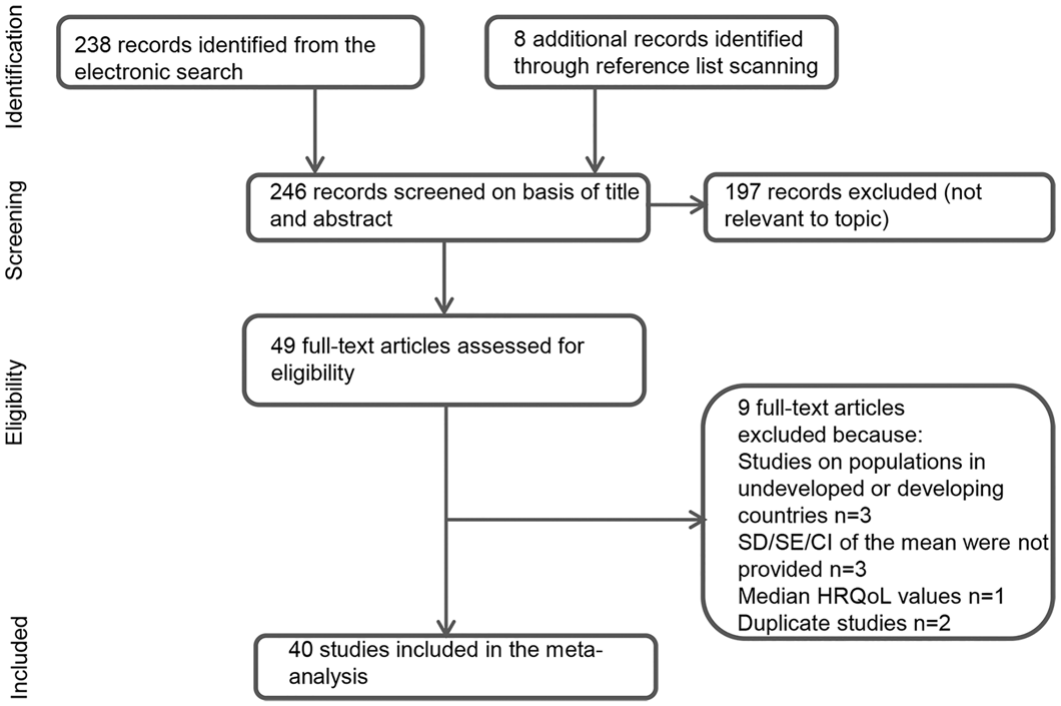
PRISMA flow diagram summarizing the study selection process. HRQoL, health-related quality of life; SD, standard deviation; SE, standard error; CI, confidence interval.

The process of study selection and extraction is presented in a PRISMA flow chart (Fig 1). A total of 40 eligible studies representing over 30,575 patients were identified after the inclusion and exclusion criteria were applied. A list of all potentially-eligible studies with reasons for exclusion is presented in S3 Appendix. The main characteristics of these studies are detailed in Table 1 [1–4,9–11,16,21–52]. The studies were published between 1991 and 2014, with most of them between 2005 and 2010. Of the 40 studies included, 10 referred to the UK, 8 to the US, 6 to the multiple country settings, 4 to Germany, 3 to Canada and Finland, 2 to Norway and Sweden, and 1 to each of Australia and Korea. Various study designs were implemented, ranging from randomised clinical trial to different observational study designs. Of the HRQoL values present in those 40 studies, 31% were observed in patients with stable angina and 29% in patients with post-ACS. The remaining 40% of the HRQoL values were associated with patients suffering from any form of CHD (including stable angina and post-ACS). The majority of patients were men (71%) with an average age of 65.35 years. There was large variation in patients’ characteristics, such as the presence and prevalence of various comorbidities (e.g. prevalence of diabetes was in range 7–38%), and treatment under evaluation (e.g. surgical procedures, pharmacological treatment or cardiovascular rehabilitation) across the studies. When reported, the time of HRQoL assessment from disease onset or treatment initiation was between 4 months and 10 years.

**Table 1:** Summary of studies reporting preference-based values in developed countries in coronary heart disease.

Author (Year), Country	Study design	CHD subgroup	Study sample (Sample size)	Instrument	Age	HRQoL value (SE)	Time (months)*	Men (%)	Diabetics (%)
Al Ruzzeh[21](2008), UK	RCT	Stable angina	Randomised to OPCAB *(n* = 66)	EQ-5D UK	64	0.6580 (0.2500)	6	84	21
			Randomised to CABG-CPB (*n* = 55)		64	0.6540 (0.2100)		83	24
Ascione (2004), UK[22]	RCT	ACS	Patients with previous MI or requirement for bypass surgery randomised to CABG-CPB (*n* = 151)	EQ-5D UK	64	0.8200 (0.0230)	36	81	33
			Patients with previous MI or requirement for bypass surgery randomised to OPCAB (*n* = 164)	EQ-5D UK	66	0.8100 (0.0187)	36	83	43
Bakhai[23](2012), France, Spain, UK	Prospective observational cohort study	ACS	Patients undergoing PCI (*N* = 1140)	EQ-5D*	62	0.8100 (0.0071)	12	78	NA
Bohmer[24](2011), Norway	Open RCT	ACS	Randomised to early invasive: angiography and PCI (*n* = 134)	15D	61	0.8890 (0.0138)	7	80	6
			Randomised to late invasive: angiography and PCI (*n* = 132)		62	0.8720 (0.0158)		71	8
Burstrom[25](2001), Sweden	Retrospective cross-sectional survey	Stable angina	Respondents with self-reported angina (*N* = 191)	EQ-5D UK	NA	0.7000 (0.0180)	NA	NA	NA
			Respondents with self-reported angina (*N* = 180)	RS	NA	0.6900 (0.0150)	NA	NA	NA
Chong[26](2009), Australia	Cross-sectional survey (prisoner population)	Stable angina	Respondents with self-reported angina (*n* = 81)	SF-6D	NA	0.6440 (0.0146)	NA	NA	NA
			Respondents with self-reported angina & MT (*n* = 17)			0.6200 (0.0410)			
Cohen[27](2011), Europe and North America	Prospective substudy as part of RCT	chd	Randomised to CABG (*n* = 810)	EQ-5D US	66	0.8470 (0.0054)	6	79	39
			Randomised to PCI (*n* = 815)		66	0.8610 (0.0052)		76	38
Denvir[28](2006), UK	Prospective observational study	chd	Patients from high SES group undergoing PCI (*n* = 876)	EQ-5D*	62	0.7500 (0.0088)	12	69	10
			Patients from low SES group undergoing PCI (*n* = 462)		62	0.6300 (0.0140)		63	13
Dunning (2008), UK[29]	Prospective cross-sectional	chd	Patients undergoing CABG (N = 621)	EQ-5D*	71	0.7000 (0.0123)	120	NA	NA
Ellis[30](2005), US	Cross-sectional survey	ACS	Patients with history of ACS (*N* = 490)	EQ-5D US	66	0.8100 (0.0081)	6	71	NA
Fryback[31](1993), US	Longitudinal cohort study	Stable angina	Respondents with self-reported angina (*n* = 68)	QWB	64	0.6600 (0.0015)	12	NA	NA
		ACS	Respondents with self-reported ACS (*n* = 20)		64	0.6400 (0,0175)			
Garster[1](2009), US	Cross-sectional random-digit-dialled survey	chd	Respondents with self-reported CHD not taking chest pain MT (*n* = 265)	EQ-5D US	70	0.8200 (0.0092)	NA	57	30
			Respondents with self-reported CHD currently taking chest pain MT (*n* = 218)		69	0.7400 (0.0142)		49	47
			Respondents with self-reported CHD not taking chest pain MT (*n* = 265)	HUI3	70	0.7500 (0.0154)		57	30
			Respondents with self-reported CHD currently taking chest pain MT (*n* = 218)		69	0.5600 (0.0237)		49	47
			Respondents with self-reported CHD not taking chest pain MT (*n* = 265)	SF-6D	70	0,7500 (0,0080)		57	30
			Respondents with self-reported CHD currently taking chest pain MT (*n* = 218)		69	0.6700 (0.0102)		49	47
			Respondents with self-reported CHD not taking chest pain MT (*n* = 265)	QWB	70	0.5800 (0.0086)		57	30
			Respondents with self-reported CHD currently taking chest pain MT (*n* = 218)		69	0.5200 (0.0095)		49	47
			Respondents with self-reported CHD not taking chest pain MT (*n* = 265)	HUI2	70	0.8000 (0.0203)		57	30
			Respondents with self-reported CHD currently taking chest pain MT (*n* = 218)		69	0.6900 (0.0277)		49	47
			Respondents with self-reported CHD not taking chest pain MT(*n* = 265)	HALex	70	0.6800 (0.0275)		57	30
			Respondents with self-reported CHD currently taking chest pain MT(*n* = 218)		69	0.5000 (0.0289)		49	47
Griffin[32](2007), UK	Prospective observational study	chd	Patients undergoing CABG (*n* = 100)	EQ-5D UK	65	0.6600 (0.0310)	72	78	13
			Patients undergoing PCI (*n* = 108)		65	0.6500 (0,0289)			
			Patients receiving MT (*n* = 131)		65	0.6100 (0.0262)			
Kattainen[33](2005), Finland	Longitudinal observational study	chd	Patients undergoing CABG (*n* = 393)	15D	63	0.8580 (0.0004)	6	73	20
			Patients undergoing PCI (*n* = 153)		61	0.8240 (0,0007)		67	
Kiessling[9](2005), Sweden	Prospective RCT	chd	Patients with CAD randomised to CML GP attending seminars—supported lipid-lowering strategy (*n* = 45)	EQ-5D UK	65	0.8000 (0.0042)	24	82	11
			Patients with CAD randomised to CML GP following local guidelines—supported lipid-lowering strategy (*n* = 43)		64	0.7600 (0.0070)		88	14
			Patients with CAD randomised to CML specialist group—supported lipid-lowering strategy (*n* = 167)		61	0.7600 (0.0014)		74	16
Kim[2](2005), UK	RCT	ACS	Patients randomised to maximal MT plus early coronary arteriography with possible myocardial revascularization (*n* = 806)	EQ-5D UK	63	0.7520 (0.0090)	12	61	15
			Patients randomised to maximal MT plus ischemia- or symptom-provoked angiography and revascularization (*n* = 820)		62	0.7360 (0.0100)		64	12
Kramer[10](2012), Germany	Quasi-experimental design	chd	CHD patients undergoing developers treatment pathway (*n* = 128)	EQ-5D Europe	69	0.7812 (0.0153)	6	79	NA
			CHD patients undergoing users treatment pathway (*n* = 70)		69	0.6936 (0.0251)		61	NA
			CHD patients undergoing controls treatment pathway (*n* = 92)		71	0.6645 (0.0265)		59	NA
Lacey[34](2003), UK	Retrospective longitudinal survey	ACS	Post-MI patients (*N* = 222)	EQ-5D UK	63	0.7180 (0.0163)	12	75	NA
Lee (2014), Korea[35]	Cross-sectional survey	chd	Respondents with self-reported CHD (*N* = 708)	EQ-5D Korea	64	0.831 (0.0090)	82	53	27
Loponen[36](2009), Finland	Prospective observational study	Stable angina	Patients undergoing CABG (*n* = 213)	15D	67	0.8579 (0.0075)	6	79	26
			Patients undergoing PCI (*n* = 208		65	0.8456 (0.0073)		69	18
Nichol (1996), Canada[37]	Observational survey-based study	Stable angina	Respondents undergoing elective cardiac catheterization (n = 41)	SG	58	0.8300 (0.0422)	NA	87	NA
Norris[3](2008), Canada	Prospective longitudinal cohort study	chd	Women undergoing catheterization (*n* = 479)	EQ-5D*	67	0.8000 (0.0046)	12	0	21
			Men undergoing catheterization (*n* = 1727)		65	0.9000 (0.0024)	12	100	22
Nowels[4](2005), US	Cross-sectional study	ACS	Post-MI patients (CCSG class I) (*n* = 67)	EQ-5D UK	65	0.7800 (0.0244)	6	69	NA
			Post-MI patients (CCSG class II) (*n* = 17)		65	0.7200 (0.0289)			
Pettersen[38](2008), Norway	Cohort study survey-based	ACS	Post-MI patients with LVEF>50% (*n* = 160)	EQ-5D UK	64	0.8300 (0.0142)	30	71	4
			Post-MI patients with LVEF = 40–50% (*n* = 53)		65	0.7200 (0.0371)			
			Post-MI patients with LVEF<40%) (*n* = 30)		66	0.7600 (0.0256)			
Ose[11](2012), Austria, Belgium, UK, France, Germany, Netherlands, Slovenia and Switzerland	Cross-sectional observational study	chd	CHD patients (*n* = 2656)	EQ-5D Europe	68	0.7300 (0.0043)	NA	70	NA
Puskas[39](2004), US	RCT	Stable angina	Randomised to OPCAB (*n* = 77)	EQ-5D UK	63	0.7900 (0.0285)	12	78	33
			Randomised to CABG (*n* = 79)		64	0.8040 (0.0259)		77	33
Saarni[40](2006), Finland	Survey-based, stratified cluster, sampling design	chd	Respondents with self-reported CHD (*n* = 555)	15D	70	0.8210 (0.0050)	NA	53	NA
			Respondents with self-reported CHD (*n* = 555)	EQ-5D UK	70	0.6840 (0.0120)			
Schweikert[41](2009), Germany	Observational survey-based study	ACS	Patients with history of MI (*N* = 2950)	EQ-5D UK	68	0.8650 (0.0028)	109	79	NA
Schweikert[42](2009), Germany	Comprehensive cohort design	ACS	Patients undergoing CR (inpatient setting) (*n* = 100)	EQ-5D Europe	58	0.8910 (0.0183)	12	79	17
			Patients undergoing CR (outpatient setting) (*n* = 47)		55	0.9410 (0.0257)		76	14
Serruys[43](2001), the ARTS (multicentre 19 countries)	RCT	Stable angina	Randomised to PCI (*n* = 593)	EQ-5D UK	62	0.8600 (0.0066)	6	77	19
			Randomised to CABG (*n* = 579)		62	0.8600 (0.0062)		76	16
Shah[44](2009), US	Observational survey-based study	ACS	Patients (70%) undergoing PCI (*N* = 32)	EQ-5D US	89	0.7800 (0.0071)	14	38	17
Sharples[45](2007), UK	RCT	Stable angina	Patients with suspected or known CAD undergoing angiography (*N* = 898)	EQ-5D UK	62	0.8020 (0.0035)	6	69	13
			Patients with suspected or known CAD undergoing angiography (*N* = 898)	SF-6D	62	0.6425 (0,0012)			
Shrive[46](2007), Canada	Prospective longitudinal cohort study	chd	Patients (70%) undergoing PCI (*N* = 1954)	EQ-5D US	NA	0.8700 (0.0034)	12	77	15
			Patients (70%) undergoing PCI (*N* = 1954)	EQ-5D UK	NA	0.8300 (0.0045)			
Stafford (2011), UK[47]	Cross-sectional survey	Stable angina	Respondents with self-reported angina (*n* = 717)	EQ-5D UK	NA	0.7110 (0.0265)	NA	NA	NA
		ACS	Respondents with self-reported MI (*n* = 550)	EQ-5D UK	NA	0.6360 (0.0171)	NA	NA	NA
Sullivan[16](2006), US	Survey-based, stratified cluster design	ACS	Respondents with self-reported MI (*n* = 244)	EQ-5D US	62	0.7040 (0.0168)	NA	NA	NA
		Stable angina	Respondents with self-reported angina (*n* = 228)		69	0.6950 (0.0201)			
Tsevat (1991), US[48]	Observational survey-based study	ACS	Survivors of MI (*N* = 80)	TTO	61	0.8700 (0.0026)	12	79	NA
Visser[49](1994), UK	Comparative study	Stable angina	Angina patients (NYHA I) receiving MT against chest pain (*n* = 10)	QWB	65	0.6800 (0.0316)	NA	73	NA
			Angina patients (NYHA II) receiving MT against chest pain (*n* = 25)		66	0.6200 (0.0180)			
			Angina patients (NYHA III) receiving MT against chest pain (*n* = 21)		67	0.6200 (0.0262)			
Weintraub (2008), US and Canada[50]	RCT	Stable angina	Randomised to PCI (*n* = 701)	SG	63	0.9300 (0.0064)	6	85	32
			Randomised to MT (*n* = 665)	SG	63	0.9300 (0.0058)	6	85	35
Werdan[51](2012), Germany	Non-interventional, multicentre open-label prospective study	Stable angina	Angina patients receiving MT (ivabradine) (*n* = 2330)	EQ-5D UK	66	0.8270 (0.0041)	4	59	33
Winkelmayer[52](2006), UK, Ireland and Netherlands	Prospective and cross-sectional design as a part of RCT	ACS	MI patients receiving MT (pravastatin) (*N* = 546)	HUI3	75	0.7350 (0.0111)	NA	48	11

* The time point of measuring HRQoL relative to the disease onset or treatment application

HRQoL, quality of life; SE, standard error; ACS, acute coronary syndrome; CHD, coronary heart disease; HALex, Health and Activity Limitation Index; HUI, health utility index; QWB, quality of well-being; RS, rating scale; SG, standard gamble; TTO, time trade-off; UK, United Kingdom; US, United States; RCT, randomized clinical trial; NA, not available; MI, myocardial infarction; MT, medical treatment; CABG, coronary artery bypass graft; CPB, cardiopulmonary bypass; OPCAB, off-pump coronary artery bypass; SES, socio-economic status; CAD, coronary artery disease; CML, case method learning; CR, cardiac rehabilitation; GP, general practitioner; PCI, percutaneous coronary intervention; CCSG; Canadian Cardiovascular Society Classification for Angina Pectoris; LVEF; left ventricular ejection fraction

The most commonly applied instrument for measuring HRQoL values was the EQ-5D (63.5%), while 15D, QWB, SF-6D, HUI, SG, TTO, RS and HALex were less prevalent (7.7, 7.7, 5.8, 5.8, 3.8, 1.9, 1.9 and 1.9%, respectively). The values measured with the EQ-5D instrument varied by the TTO “tariffs” utilized (i.e. UK, US, Europe and Korea). The scoring of most of the EQ-5D values was based on the UK “tariff” (53%) while US, European and Korean “tariff” were less present (19, 8, 8 and 3%, respectively). Three studies provided no explicit information on the EQ-5D scoring “tariff” used, however, in order to allow for the evidence synthesis these values were grouped together with the UK “tariffs”. The values measured with the HUI instrument were presented in the studies as both mark 2 (i.e. HUI2) and mark 3 (i.e. HUI3).

Finally, the correlation coefficients between the instrument-specific HRQoL values, necessary for conducting a multivariate meta-analysis on the data set formed, were reported in only one of the studies included in the data set [31]. Therefore, some of the correlation coefficients were retrieved from other studies on cardiovascular patients or general populations without severe comorbidities (S1 Table) [31,53,54–57]. Nevertheless, a great number of within the instrument-specific correlation coefficients remained missing.

### Multivariate meta-analysis estimates in post-ACS, stable angina, and general CHD

Table 2 summarizes the instrument-specific estimates synthesized through multivariate meta-analysis in the post-ACS, stable angina subgroups, and general CHD assessed on the full data set. The values for estimates synthesized in post-ACS ranged from 0.64 (QWB) to 0.92 (EQ-5D European”tariff”), while in stable angina estimates ranged from 0.64 (SF-6D) to 0.89 (SG). In general CHD, the values ranged from 0.60 (HALex) to 0.89 (SG). Between-study SDs and variance-covariance matrices for HRQoL in the post-ACS and stable angina subgroups, and general CHD, when these parameters could be estimated, are reported in S2–S4Tables.

**Table 2:** Post-acute coronary syndrome, stable angina, and general CHD parameter estimates for HRQoL and multivariate heterogeneity statistics using multivariate meta-analysis.

Instrument	N	Post-ACS subgroup	$I_{H}^{2}$	$I_{R}^{2}$	N	Stable angina subgroup	$I_{H}^{2}$	$I_{R}^{2}$	N	CHD (full dataset)	$I_{H}^{2}$	$I_{R}^{2}$
**15D**	1	**0.8816** (0.0074)			1	**0.8515** (0.0037)			4	**0.8495** (0.0069)		99.9%
**EQ-5D Europe**	1	**0.9170** (0.0105)							3	**0.7915** (0.0625)		99.7%
**EQ5D Korea**									1	**0.8310** (0.0090)		
**EQ-5D UK**	8	**0.7638** (0.0246)		99.3%	7	**0.7792** (0.0250)		99.4%	22	**0.7591** (0.0122)		99.7%
**EQ-5D US**	3	**0.7662** (0.0308)		97.3%	1	**0.6950** (0.0201)			7	**0.8012** (0.0128)		98.0%
**HALex**									1	**0.5967** (0.0075)		
**HUI2**									1	**0.7626** (0.0061)		
**HUI3**	1	**0.7350** (0.0111)							2	**0.7259** (0.0118)		63.5%
**QWB**	1	**0.6400** (0.0175)			2	**0.6517** (0.0097)		97.5%	4	**0.6287** (0.0189)		99.4%
**RS**									1	**0.6900** (0.0150)		
**SF-6D**					2	**0.6413** (0.0017)		51.9%	3	**0.6859** (0.0131)		99.3%
**SG**					2	**0.8889** (0.0492)		99.9%	2	**0.8889** (0.0492)		99.9%
**TTO**	1	**0.8700** (0.0026)							1	**0.8700** (0.0026)		
			86.8%	70.7%			68.1%	92.7%			91.2%	93.9%

N presents the number of instrument-specific HRQoL values used for estimation.

All model coefficients with the level of significance p < 0.001 are presented in bold.

Standard errors of parameter estimates are showed in parentheses.

ACS, acute coronary syndrome; CHD, coronary heart disease; HRQoL, health-related quality of life; UK, United Kingdom; US, United States; HALex, Health and Activity Limitation Index; HUI, health utility index; QWB, quality of well-being; RS, rating scale; SG, standard gamble; TTO, time trade-off.

In this evidence synthesis, some of the instrument-specific HRQoL values included in the data set were present only as single inputs. Notably, the output of the multivariate meta-analysis in the aforementioned cases reflected the initial instrument-specific HRQoL inputs. Because the EQ-5D UK”tariff” values were the only instrument-specific values available as multiple inputs in both post-ACS and stable angina subgroups, a relatively fair comparison of the level of summarized HRQoL between the two subgroups would only be possible for this instrument-specific subgroup. This comparison indicated slightly lower estimates in post-ACS (i.e. 0.76) than the ones in stable angina (i.e. 0.78).

Large variations are noticeable across instrument-specific estimates of HRQoL in CHD presented in Table 2. The highest level of HRQoL was observed for the SG, TTO and 15D estimates, which were followed by slightly lower EQ-5D and HUI estimates, while the RS, SF-6D, QWB and HALex were the instruments with lowest HRQoL estimates.

Interestingly, substantial unexplained between-study but within-instrument heterogeneity was observed in all the multivariate meta-analysis models with the most excessive levels of heterogeneity observed in general CHD assessed on the full dataset (Table 2). There was a general agreement between both $I_{H}^{2}$ and $I_{R}^{2}$ statistics on the level of heterogeneity.

### Regression analysis

The EQ-5D UK “tariff” values were the only instrument-specific values available with more than 10 observations per instrument used and therefore in line with our requirement for regression analysis. This led to reducing the analysis from a multivariate to a univariate meta-regression. Table 3 presents the EQ-5D UK “tariff” estimates synthesized through a univariate meta-regression where the impact of disease subgroup, age, publication year, and prevalence of diabetes and of men was examined on the HRQoL. Here, increasing age was found to generally reduce the level of HRQoL while prevalence of diabetes and higher proportion of men increased its level. The impact of publication year was inconclusive. However, none of the covariates examined was found to provide a significant improvement in the model fit nor did they reduce the between-study heterogeneity (Table 3).

**Table 3:** Parameter estimates and heterogeneity statistics for the EQ-5D UK “tariff” estimates using univariate meta-regression.

Model number	Model	Post-ACS	*I*^2^	Model number	Angina	*I*^2^	Model number	General CHD	*I*^2^
*1*	*b*_0_	**0.7587** (0.0215)	99.6%	*5*	**0.7542** (0.0178)	99.5%	*9*	**0.7652**(0.0165)	99.6%
	*b*_*Disease type*_	0.0165 (0.0339)			0.0512 (0.0396)				
	*b*_*Age*_	-0.0051 (0.0059)			-0.0035 (0.0057)			-0.0056 (0.0057)	
*2*	*b*_0_	**0.7588** (0.0347)	99.7%	*6*	**0.7497** (0.0355)	99.6%	*10*	**0.7604** (0.0326)	99.7%
	*b*_*Disease type*_	0.0059 (0.0333)			0.0270 (0.0348)				
	*b*_*Publication year*_	-0.0002 (0.0052)			0.0005 (0.0051)			-0.0001 (0.0050)	
*3*	*b*_0_	**0.7275** (0.0510)	99.7%	*7*	**0.7405** (0.0443)	99.7%	*11*	**0.7340** (0.0426)	99.7%
	*b*_*Disease type*_	0.0125 (0.0457)			0.0306 (0.0450)				
	*b*_*Diabetes*_	0.0029 (0.0024)			0.0017 (0.0026)			0.0027 (0.0022)	
*4*	*b*_0_	**0.5982** (0.1456)	99.7%	*8*	**0.5572** (0.1325)	99.7%	*12*	**0.5964** (0.1407)	99.7%
	*b*_*Disease type*_	0.0028 (0.0325)			0.0540 (0.0331)				
	*b*_*Men*_	0.0024 (0.0020)			0.0028 (0.0018)			0.0025 (0.0019)	

All model coefficients with the level of significance p < 0.001 are presented in bold.

Standard errors of parameter estimates are showed in parentheses.

For regression models 1–4, an intercept is provided assigned with *b*_0_, coefficients *b*_*Disease type*_ (referring to post-ACS) and in model 1 *b*_*Age*_, in model 2 *b*_*Publication year*,_ in model 3 *b*_*Diabetes*_ and in model 4 *b*_*Men*_. The number of EQ-5D values in post-ASC available for models 1–4 was 8.

For regression models 5–8, an intercept is provided assigned with *b*_0_, coefficients *b*_*Disease type*_ (referring to stable angina) and in model 5 *b*_*Age*_, in model 6 *b*_*Publication year*,_ in model 7 *b*_*Diabetes*_ and in model 8 *b*_*Men*_. The number of EQ-5D values in stable angina available for models 5–8 was 7.

For regression models 9–12, an intercept is provided assigned with *b*_0_, coefficients in model 9 *b*_*Age*_, in model 10 *b*_*Publication year*,_ in model 11 *b*_*Diabetes*_ and in model 12 *b*_*Men*_. The number of EQ-5D values in CHD in general available for models 19–12 was 22.

Example for interpretation: Model 4 –summary EQ-5D UK “tariff” estimate in men with post-ACS would be 0.6034 (i.e 0.5982+0.0028+0.0024), and 0.601 in women with similar characteristics (0.5982+0.0028).

ACS, acute coronary syndrome; CHD, coronary heart disease; UK, United Kingdom; *b*_0_, intercept.

Sensitivity analysis on the correlation coefficients between instrument-specific values was undertaken. The result of this sensitivity analysis was that the model was overall robust to different values of the correlation coefficients (S5 Table). Moreover, the SEs of instrument-specific estimates were generally insensitive to ignoring the correlation between the instrument-specific values or setting the values of correlation coefficients to 0.5.

## Discussion

This is the first study that systematically summarized and synthesized instrument-specific preference-based HRQoL values in CHD, and its underlying disease-forms, post-ACS and stable angina in developed countries. Pooled mean HRQoL values were estimated and a large variation was observed both within and between the instrument-specific values. This variation could be explained by the large underlying heterogeneity in the study populations, and the observed and unobserved variation between the HRQoL instruments. Other factors possibly include the impact of treatment applied, initial (acute) disease severity level, national and socio-economic characteristics, various comorbidities present or the time of assessment. Moreover, the fact that TTO “tariffs” derived in nationality-specific population samples for the preference-based scoring of the EQ-5D vary across countries [58], suggests possible larger variations in the HRQoL values in CHD patients from various national or multinational settings. Additional arguments emphasize that unobservable differences in cultural or socio-economic status may also be present in representatives of general population samples selected for the assessment of tariffs [59]. The consequence of this would then be a greater underlying variability in HRQoL values in CHD assessed with instruments utilizing those tariffs. In essence, the aforementioned concerns may have a direct impact on the generalizability of country-specific HRQoL values to various national or multinational settings.

The regression analysis indicated no significant association between available study-level covariates and HRQoL estimates. Caution is needed in the interpretation of those findings, especially when low power for testing the effect of study-level covariates on the pooled HRQoL estimate is present—namely, only 22 EQ-5D UK “tariff” values were available for the regression-analysis. However, the reduction and increase in HRQoL observed with advancing age and higher proportion of men, respectively is in line with other published information [60]. Surprisingly, studies with a higher proportion of patients with diabetes had a higher average HRQoL estimate what contrasts the finding by Xie et al. [61]. This may be due to the missing information of the prevalence of diabetes across the studies as well as an example of ecological fallacy.

The variation between the instruments in the HRQoL estimates observed in our study is in agreement with the findings from other studies that demonstrated the differences across instrument-specific HRQoL values [62–64](Fryback et al 2009)[65]. Similarly to the study by Johnson et al, we observed higher levels of HRQoL when summarizing the EQ-5D US “tariffs” compared to the UK “tariffs”[58]. Additionally, SG values commonly exceed TTO values and RS values, a finding that was also confirmed in our study [66,67]. Furthermore, our synthesized HUI3 estimates in CHD were lower compared to the EQ-5D estimates, but similar to the findings of O’Brien et al. in patients at increased risk of sudden cardiac death and receiving implantable defibrillator therapy[63]. In our study, both the summarized and study-level SF-6D values were lower than EQ-5D values in CHD what contrasts the observations of Brazier et al.[1,45]. We observed the lowest level of HRQoL in CHD for estimates measured with the QWB and the HALex, what is in line with the patterns of values in general US population measured with six preference-based instruments by Fryback et al.[65] Differences such as the valuation technique, bounds of scale and sensitivity to change after treatment are only some of potential reasons for the variation between the instrument-specific HRQoL estimates [62–64].

For the evidence synthesis we applied multivariate meta-analysis given that when information on various instrument-specific values is sparse, it allows for “borrowing of strength” from the values available by accounting for the correlation between them [12,13]. Though such an approach may provide more precise summarized estimates [12,13] than a meta-analysis where the correlation is ignored, this did not hold in our study due to a high between-study variation.

Potentials for direct comparisons of our analysis to other synthesized HRQoL values are limited due to the lack of studies meta-analysing preference-based values in CHD. A meta-analysis of 84 studies identified to address HRQoL in cardiac patients by Kinney et al. may be one potential comparator to our study [68]. Kinney et al. investigated the effect of pharmacological, surgical, nursing or other treatment on HRQoL and found a small positive effect of treatment (i.e. standardised mean difference (*d*) = 0.31). Despite certain similarity in the patient populations investigated between the two studies can be acknowledged, numerous differences such as the study inclusion criteria (i.e. any measurement of HRQoL including the ones of single health attributes), choice of study effect size (i.e. standardised mean difference), the period of data collection (i.e. 1987–1991) and the methodology used for conducting meta-analysis (i.e. fixed-effect model) hamper adequate comparisons.

Another comparator to our study may be a review by Dyer et al. on the EQ-5D values in cardiovascular disease (CVD)[69]. Dyer et al. summarized and stratified the EQ-5D values across different CVD subgroups (e.g. ischemic heart disease (IHD) such as angina/myocardial infarction/CHD, heart failure etc.) and, when feasible, across three severity level categories defined by the percentage of patients in a given group in class III/IV of NYHA or CCS class[69]. Their stratification of the EQ-5D values with IHD collected at baseline resulted in the range of values from 0.45 for moderate/severe angina to 0.80 for mild angina. The authors did not synthesise these values across different severity levels due to the high heterogeneity observed (i.e. *I*^2^ of 82–96%), but suggested more rigorous study inclusion criteria and the possibility to expand their data set with more recent publications (i.e. studies published after 2008) as a method to reduce some of the heterogeneity observed[69]. However, the more rigorous inclusion criteria that our study proposed such as the inclusion of only mean HRQoL values measured in patients in stable or post-acute disease state, and incorporating HRQoL values from a wider publication range (i.e. 1990–2014) in the data set, did not reduce the high heterogeneity observed across studies. Notably, the heterogeneity indices observed in the study by Dyer et al. and the ones observed between the EQ-5D values in our study cannot be directly compared[69]. Differences between the two data sets and the method for disease stratification (i.e. post-ACS and stable angina subgroups vs. CCS class categories reported in ICH) are limiting such a comparison.

Our analysis is confronted with certain limitations. The main limitation of our study is that we analysed study-level and not patient-level data. Analysing patient-level data might provide significant improvements in our analysis. If detailed information on patients currently nonclassified specific CHD from was available, this would allow for a more accurate disease-specific allocation of HRQoL values. Conducting the multivariate meta-analysis on such a data set could possibly lead to estimates with lower level of between-study heterogeneity. Another limitation of our study was that we conducted the meta-regression analysis only on the subset of the EQ-5D UK “tariff” values due to a relatively small number of studies providing other instrument-specific values. This regression analysis was also limited with the respect to the variety of covariates investigated. Covariates such as patients’ socioeconomic status, presence of comorbidities other than diabetes or the impact of treatment applied were not investigated in the regression analysis due to their scarce information across the studies [70]. Expectedly, this study was confronted with the missing information on within-study correlation coefficients. This was solved by retrieving the correlation coefficients reported in other studies on cardiovascular patients or general populations. The sensitivity of the study results on the correlation coefficients utilized was tested in the sensitivity analysis. Furthermore, the publication bias was not formally assessed using a funnel plot due to the small number of instrument-specific HRQoL values included in this study as well as considerable heterogeneity. Also, publication bias is not expected in our meta-analysis given that the HRQoL values are commonly measured as secondary study outcomes and, therefore, are not expected to impact the decision to publish. Finally, information on HRQoL in CHD measured with non-preference-based and disease-specific instruments was not included in our analysis.

Importantly, our study did not aim to investigate what the most appropriate and reliable instrument to measure HRQoL in CHD is, but rather to summarize and synthesize all the available evidence of preference-based instrument-specific values. The decision on the most robust instrument-specific estimate to be applied in a CUA depends not only on the appropriateness and reliability of an instrument to measure HRQoL in CHD but also on its agreement with instrument-specific values available for other health states modelled in the CUA. Furthermore, some decision-makers might argue that considering the previously discussed reasons for country- and centre-specific variability in HRQoL values, one should simply choose a single country-specific and CHD-form specific HRQoL value. However, in the case where multiple country-specific values exist or even a single such value is unavailable, the decision on the most robust value becomes more complex and needs to rely on an evidence-synthesis exercise Moreover, although distinguishing between underlying CHD-forms seems as more clinically relevant, CUAs in both primary and secondary prevention of CHD often model a general CHD health state [71–74]. An evidence synthesis of all available HRQoL values may again be considered to select a robust HRQoL estimate in CHD. Such an estimate could then reflect more appropriately the complex nature of CHD and its various manifestations. This motivated us to consider an evidence-synthesis of HRQoL values in overall CHD.

Finally, characterizing the between study heterogeneity not only provides a better mean estimate for HRQoL values used in CUA but it also provides a better understanding on the uncertainty around the HRQoL value and how this translates into uncertainty around the CUA outcomes. This uncertainty, as we showed in our study, is considerable and as it is mostly found between studies, it is ignored when a single value from an individual study is selected. Dias et al. proposed that in the presence of between-study heterogeneity, using the predictive distribution is the appropriate way to characterize parameter uncertainty when embedding synthesized evidence in CUA [75]. Researchers using the findings from this study for economic evaluation purposes will therefore have to rely in generating values that incorporate both within- and between-study standard deviation (predictive distribution) provided in the results section of this article.

## Conclusions

This study represents the first evidence synthesis of instrument-specific preference-based HRQoL values in post-ACS, stable angina, and CHD in general. Considerable differences in mean HRQoL estimates were observed both within and between the instruments. These differences characterized by large between-study heterogeneity may be explained by both the observed and unobserved methodological differences across instruments and underlying study-level characteristics. Current CUAs in CHD ignore this between-study study heterogeneity. Therefore, multivariate meta-analysis can facilitate quantifying this heterogeneity for HRQoL estimates and offer the means for uncertainty around HRQoL values to be translated to uncertainty in economic models.

## Supporting Information

S1 Appendix PRISMA checklist. (DOC)

S2 Appendix Search strategy for MEDLINE and EMBASE. (DOCX)

S3 Appendix Potential studies excluded. (DOCX)

S1 Table Correlation coefficients between the HRQoL values assessed with different instruments. (DOCX)

S2 Table Between-study SDs and variance-covariance matrix in post-ACS model. (DOCX)

S3 Table Between-study SDs and variance-covariance matrix in stable angina model. (DOCX)

S4 Table Between-study SDs and variance-covariance matrix in CHD model. (DOCX)

S5 Table Parameter estimates and multivariate heterogeneity statistics in the sensitivity analysis on different correlation coefficients between HRQoL instruments in CHD. (DOCX)
