# Supplementary material for: Multivariate Meta-Analysis of Preference-Based Quality of Life Values in Coronary Heart Disease
Source: PLoS One. 2016 Mar 24;11(3):e0152030. doi: 10.1371/journal.pone.0152030 (PMC4806923; doi:10.1371/journal.pone.0152030)
Supplement: S2 Appendix — (DOCX) [file pone.0152030.s002.docx]

**S2 Appendix.** Search strategy for MEDLINE and EMBASE

("coronary artery disease"[All Fields] OR "coronary heart disease"[All Fields] OR "myocardial infarction"[All Fields] OR "angina"[All Fields] OR "acute coronary syndrome"[All Fields]) AND ("quality of life"[All Fields] OR "utility"[All Fields] OR "utilities"[All Fields] OR "Outcome Assessment (Health Care)"[Mesh] OR "quality of life"[Mesh] OR "preferences"[All Fields]) AND ("HALex"[All Fields] OR "Health Utilities Index"[All Fields] OR "quality of well-being"[All Fields] OR "QWB"[All Fields] OR "rating scale"[All Fields] OR "standard gamble"[All Fields] OR "time trade-off"[All Fields] OR "15D"[All Fields] OR "SF-6D"[All Fields] OR "EQ-5D"[All Fields] OR "EuroQol"[All Fields]) NOT (Review[ptyp] OR Editorial[ptyp] OR Letter[ptyp] OR Clinical Conference[ptyp]) AND ( ("1990/01/01"[PDAT] : "2014/11/30"[PDAT]))
