# Supplementary material for: Multivariate Meta-Analysis of Preference-Based Quality of Life Values in Coronary Heart Disease
Source: PLoS One. 2016 Mar 24;11(3):e0152030. doi: 10.1371/journal.pone.0152030 (PMC4806923; doi:10.1371/journal.pone.0152030)
Supplement: S3 Appendix — (DOCX) [file pone.0152030.s003.docx]

**S3 Appendix** Potential studies excluded

Reason – Studies on undeveloped or developing countries:

1. Ekwunife et al. Health Utilities Index Mark 3 (HUI3) demonstrated construct validity in a Nigerian population with hypertension. Quality of Life Research, 2013
2. Wu et al. Chronic stable angina is associated with lower health-related quality of life: evidence from Chinese patients. Plos one, 2014.
3. Yu et al. Long-term changes in exercise capacity, quality of life, body anthropometry, and lipid profiles after a cardiac rehabilitation program in obese patients with coronary heart disease. The American journal of cardiology, 2003.

Reason – Studies where SD/SE/CI of the mean HRQoL value of interest were not provided:

1. Hatoum et al. Comparison of the HUI3 with the SF-36 preference based SF-6D in a clinical trial setting. Value in Health, 2004.
2. Oldridge et al. Community or patient preferences for cost-effectiveness of cardiac rehabilitation: does it matter? Eur J Prev Cardiology, 2008.
3. Levin et al. Health-Related Quality of Life of Ticagrelor versus Clopidogrel in Patients with Acute Coronary Syndromes—Results from the PLATO Trial. VIH, 2013.

Reason – Studies providing median HRQoL values:

1. Van Stel et al. Comparison of the SF-6D and the EQ-5D in patients with coronary heart disease. Health Qual Life Outcomes, 2006.

Reason – Duplicate studies:

1. Legrand et al. Three-year outcome after coronary stenting versus bypass surgery for the treatment of multivessel disease. Circulation, 2004.
2. Oreopoulos et al. Association between obesity and health-related quality of life in patients with coronary artery disease. Int J Obes, 2010.
