# Supplementary material for: Multivariate Meta-Analysis of Preference-Based Quality of Life Values in Coronary Heart Disease
Source: PLoS One. 2016 Mar 24;11(3):e0152030. doi: 10.1371/journal.pone.0152030 (PMC4806923; doi:10.1371/journal.pone.0152030)
Supplement: S1 Table — (DOCX) [file pone.0152030.s004.docx]

**S1 Table. Correlation coefficients between the HRQoL values assessed with different instruments.**

| Instrument | 15D | EQ-5D UK | EQ-5D Europe | EQ-5D US | EQ-5D Korea | SF-6D | TTO | SG | HUI2 | HUI3 | QWB | RS | HALex |
| --- | --- | --- | --- | --- | --- | --- | --- | --- | --- | --- | --- | --- | --- |
| 15D | 1 |  |  |  |  |  |  |  |  |  |  |  |  |
| EQ-5D UK | 0.39(56) | 1 |  |  |  |  |  |  |  |  |  |  |  |
| EQ-5D Europe | NA | 0.99*(54) | 1 |  |  |  |  |  |  |  |  |  |  |
| EQ-5D US | NA | 0.99(54) | 0.99*(54) | 1 |  |  |  |  |  |  |  |  |  |
| EQ-5D Korea | NA | NA | NA | NA | 1 |  |  |  |  |  |  |  |  |
| SF-6D | 0.51(56) | 0.46(56) | NA | 0.72(54) | NA | 1 |  |  |  |  |  |  |  |
| TTO | 0.095(55) | -0.05(57) | NA | NA | NA | NA | 1 |  |  |  |  |  |  |
| SG | NA | -0.06(57) | NA | NA | NA | NA | 0.52(53) | 1 |  |  |  |  |  |
| HUI2 | 0.52(56) | 0.5(56) | NA | NA | NA | 0.44(56) | NA | NA | 1 |  |  |  |  |
| HUI3 | 0.44(56) | 0.61(56) | NA | NA | NA | 0.4(56) | 0.15(57) | 0.16(57) | 0.77(56) | 1 |  |  |  |
| QWB | 0.53(56) | 0.44(56) | NA | NA | NA | 0.43(56) | 0.41(31) | NA | 0.47(56) | 0.47(56) | 1 |  |  |
| RS | NA | 0.39(57) | NA | NA | NA | NA | 0.46(53) | 0.4(53) | NA | 0.41(57) | NA | 1 |  |
| HALex | NA | NA | NA | NA | NA | NA | NA | NA | NA | NA | NA | NA | 1 |

*Correlation coefficients assumed to be the same as the ones between the values assessed with EQ-5D UK and other instruments.

HRQoL, health related quality of life; UK, United Kingdom; US, United States; HUI, health utility index; QWB, quality of well-being; RS, rating scale; SG, standard gamble; TTO, time trade-off; HALex, Health and Activity Limitation Index.
