# Supplementary material for: Multivariate Meta-Analysis of Preference-Based Quality of Life Values in Coronary Heart Disease
Source: PLoS One. 2016 Mar 24;11(3):e0152030. doi: 10.1371/journal.pone.0152030 (PMC4806923; doi:10.1371/journal.pone.0152030)
Supplement: S2 Table — (DOCX) [file pone.0152030.s005.docx]

**S2 Table.** **Between-study SDs and variance-covariance matrix in post-ACS model.**

| Instrument | SD | Variance-Covariance matrix | |
| --- | --- | --- | --- |
|  |  | EQ-5D UK | EQ-5D US |
| EQ-5D UK | 0.07 | - |  |
| EQ-5D US | 0.05 | -0.09 | - |

ACS, acute coronary syndrome; SD, standard deviation; UK, United Kingdom; US, United States.
