# Supplementary material for: Multivariate Meta-Analysis of Preference-Based Quality of Life Values in Coronary Heart Disease
Source: PLoS One. 2016 Mar 24;11(3):e0152030. doi: 10.1371/journal.pone.0152030 (PMC4806923; doi:10.1371/journal.pone.0152030)
Supplement: S3 Table — (DOCX) [file pone.0152030.s006.docx]

**S3 Table.** **Between-study SDs and variance-covariance matrix in stable angina model.**

| Instrument | SD | Variance-Covariance matrix | | |  |
| --- | --- | --- | --- | --- | --- |
|  |  | EQ-5D UK | QWB | SF-6D | SG |
| EQ-5D UK | 0.06 | - |  |  |  |
| QWB | 0.01 | 0.00 | - |  |  |
| SF-6D | 0.00 | 1.00 | 0.01 | - |  |
| SG | 0.06 | -0.01 | 0.02 | -0.01 | - |

SD, standard deviation; UK, United Kingdom; QWB, quality of well-being; SG, standard gamble.
