# Supplementary material for: Multivariate Meta-Analysis of Preference-Based Quality of Life Values in Coronary Heart Disease
Source: PLoS One. 2016 Mar 24;11(3):e0152030. doi: 10.1371/journal.pone.0152030 (PMC4806923; doi:10.1371/journal.pone.0152030)
Supplement: S4 Table — (DOCX) [file pone.0152030.s007.docx]

**S4 Table. Between-study SDs and variance-covariance matrix in CHD model.**

| Instrument | SD | Variance-Covariance matrix | | | | | | | |
| --- | --- | --- | --- | --- | --- | --- | --- | --- | --- |
|  |  | 15D | EQ-5D Europe | EQ-5D UK | EQ-5D US | HUI3 | QWB | SF-6D | SG |
| 15D | 0.02 | - |  |  |  |  |  |  |  |
| EQ-5D Europe | 0.11 | -0.18 | - |  |  |  |  |  |  |
| EQ-5D UK | 0.07 | 1,000 | -0.04 | - |  |  |  |  |  |
| EQ-5D US | 0.06 | 996.20 | 1.79 | 996.40 | - |  |  |  |  |
| HUI3 | 0.02 | 205.10 | -19.87 | 203.60 | 119.50 | - |  |  |  |
| QWB | 0.04 | 206.30 | -19.83 | 204.70 | 120.60 | 1,000 | - |  |  |
| SF-6D | 0.07 | -996.50 | 2.04 | -996.40 | -985.50 | -286.00 | -287.20 | - |  |
| SG | 0.06 | -0.37 | -0.61 | -0.04 | 0.48 | -4.34 | -4.32 | 0.31 | - |

CHD, coronary heart disease; SD, standard deviation; UK, United Kingdom; US, United States; Health and Activity Limitation Index; HUI, health utility index; QWB, quality of well-being; SG, standard gamble.
