# Supplementary material for: Multivariate Meta-Analysis of Preference-Based Quality of Life Values in Coronary Heart Disease
Source: PLoS One. 2016 Mar 24;11(3):e0152030. doi: 10.1371/journal.pone.0152030 (PMC4806923; doi:10.1371/journal.pone.0152030)
Supplement: S5 Table — (DOCX) [file pone.0152030.s008.docx]

**S5 Table. Parameter estimates and multivariate heterogeneity statistics in the sensitivity analysis on different correlation coefficients between HRQoL instruments in CHD.**

| Instrument | No correlation | Correlation coefficient = 0.5 |
| --- | --- | --- |
| 15D | **0.8495** (0.0069) | **0.8492** (0.0059) |
| EQ-5D Europe | **0.7915** (0.0626) | **0.7915** (0.0627) |
| EQ5D Korea | **0.8310** (0.0090) | **0.8310** (0.0090) |
| EQ-5D UK | **0.7591**(0.0121) | **0.7599** (0.0121) |
| EQ-5D US | **0.8011** (0.0128) | **0.7988** (0.0148) |
| HALex | **0.5926** (0.0076) | **0.5955** (0.0072) |
| HUI2 | **0.7596** (0.0062) | **0.7615** (0.0059) |
| HUI3 | **0.7258**(0.0118) | **0.7265** (0.0114) |
| QWB | **0.6287** (0.0187) | **0.6305** (0.0167) |
| RS | **0.6900** (0.0150) | **0.6900** (0.0150) |
| SF-6D | **0.6859** (0.0131) | **0.6739** (0.0115) |
| SG | **0.8889** (0.0490) | **0.8890** (0.0488) |
| TTO | **0.8700** (0.0026) | **0.8700** (0.0026) |
|  |  |  |
| $I_{H}^{2}$ | 90.4% | 91.2% |

All model coefficients with the level of significance p < 0.001 are presented in bold.

Standard errors of parameter estimates are showed in parentheses.

HRQoL, health-related quality of life; CHD, coronary heart disease; UK, United Kingdom; US, United States; HALex, Health and Activity Limitation Index; HUI, health utility index; QWB, quality of well-being; RS, rating scale; SG, standard gamble; TTO, time trade-off.
